# Pro370Leu mutant myocilin impairs mitochondrial functions in human trabecular meshwork cells

**Published:** 2009-04-23

**Authors:** Yuan He, Kar Wah Leung, Ye-Hong Zhuo, Jian Ge

**Affiliations:** 1 State Key Laboratory of Ophthalmology, Zhongshan Ophthalmic Center, Sun Yat-sen University, Guangzhou, China; 2 Department of Neural and Behavioral Sciences, Pennsylvania State University College of Medicine, Hershey, PA

## Abstract

**Purpose:**

Oxidative stress is a risk factor for the onset and progression of primary open-angle glaucoma (POAG), but the exact molecular basis remains unknown. Here, we investigated the mechanisms for Pro370Leu mutant myocilin to induce mitochondrial dysfunction and subsequent reactive oxygen species (ROS) generation in trabecular meshwork (TM) cells obtained from POAG individuals.

**Methods:**

Primary non-diseased human TM cultures were transfected with pIRES-EGFP (Mock), pIRES-wild-type (WT), or pIRES-Pro370Leu mutant myocilin. Transfection efficiency and myocilin subcellular localization were determined by polymerase chain reaction (PCR), western blot analysis, and confocal microscopy. ROS levels as well as free Ca^2+^ concentrations in cytoplasm ([Ca^2+^]_c_) and mitochondria ([Ca^2+^]m) were examined by 2’7’-dichlorofluorescein diacetate (H_2_-DCF-DA), Fluo-3 acetoxymethyl ester (Fluo-3/AM), and Rhod-2 acetoxymethyl ester (rhod-2/AM), respectively, using flow cytometry. Mitochondrial functions were revealed by changes in mitochondrial membrane potential (ΔΨm) and ATP production, which were found by fluorescent probe 5,5′,6,6’-tetrachloro-1,1’3,3′-tetraethylbenzimid azolocarbocyanine iodide (JC-1) and a luciferin/luciferase-based ATP assay, respectively.

**Results:**

Both WT and Pro370Leu mutant myocilin are localized in the mitochondria of TM cells as indicated using confocal microscopy and western blot analysis. Overexpression of WT myocilin decreases ΔΨm, which is further reduced by Pro370Leu mutant myocilin. TM cells that overexpressed Pro370Leu mutant myocilin have greater cell death, higher endogenous ROS, [Ca^2+^]_c_, and [Ca^2+^]_m_ levels, and lower ATP production, and yet, these effects are not seen in the overexpression of WT myocilin.

**Conclusions:**

Our findings suggested that Pro370Leu mutant myocilin causes mitochondrial defects, which may lead to TM cell dysfunction and even cell death. Therefore, preventive measures targeting mitochondrial protection may delay the onset of glaucoma in individuals carrying the Pro370Leu myocilin mutation.

## Introduction

Glaucoma is the second leading cause of irreversible blindness worldwide, which affects approximately 70 million people [1]. Primary open-angle glaucoma (POAG), the most common form of this disease, contributes to a large proportion of blindness cases in the elderly [2-6].

POAG is associated with intraocular pressure (IOP) elevation, which is caused by abnormal resistance of aqueous outflow through the trabecular meshwork (TM), a specialized tissue lining the outflow pathway of the eye [7,8]. The mechanisms that lead to such abnormalities in TM are largely unknown. Possible means include gene mutations (e.g., myocilin, optineurin) [9,10], vascular alterations [11,12], mechanical injuries [13-16], and local oxidative stress [17,18]. Elevated IOP leads to progressive optic neuropathy and ganglion cell death in the neural retina that often result in irreversible loss of vision [13-16].

Myocilin is also known as “TIGR” for trabecular meshwork-inducible glucocorticoid response. Mutation of this gene was first reported to associate with juvenile-onset open-angle glaucoma (JOAG) in 1997 [19]. Later, more than 70 mutation sites in the myocilin gene were identified to be related to the pathogenesis of approximately 3% of familial autosomal dominant adult-onset open-angle glaucoma and a greater proportion of JOAG [20]. Among the identified mutations, the Pro370Leu mutation (OMIM 601652; allelic variant 0.007) is responsible for one of the most severe glaucoma phenotypes [21-24]. These patients have high IOP, typical glaucomatous cupping of the optic disc, and a thinner nerve fiber layer and are unresponsive to standard pharmacological treatments [25]. In this study, we investigated how Pro370Leu mutant myocilin affects the TM cells.

Myocilin is a signal peptide secretary protein [26-29]. It has both intracellular and intercellular functions [30,31] and can be found in various organelles such as the endoplasmic reticulum (ER), Golgi apparatus [32-38], and mitochondria [39-42]. Myocilin is imported into the mitochondria and the specific transporter is currently unknown [42]. It localizes on inner and outer membranes as well as in the intermembranous space but not in the mitochondrial matrix [42]. Although the myocilin transcript and protein are ubiquitously expressed in multiple ocular tissues [43], its interaction with the mitochondria appears to be cell specific. Myocilin-mitochondria interaction is seen in TM cells and astrocytes but not in corneal ﬁbroblasts [40,41]. These findings prompt us to hypothesize that myocilin mutations may alter mitochondrial function in TM cells, which explains the specificity of myocilin mutations to glaucoma.

Here, we report that TM cells that overexpress Pro370Leu mutant myocilin demonstrate features of mitochondrial dysfunction including increased cellular reactive oxygen species (ROS) and decreased mitochondrial membrane potential (ΔΨm) and ATP production as well as dysfunction in calcium regulation. These findings indicate Pro370Leu mutant myocilin may increase vulnerability of TM cells to various cellular insults and cause impaired functioning.

## Methods

### Materials

All tissue culture reagents were obtained from Gibco BRL (Gaithersburg, MD). The luciferin/luciferase-based ATP assay kit was purchased from Sigma (St. Louis, MO). 2’,7’-dichlorodihydrofluorescin diacetate (H_2_-DCF-DA), 5,5′,6,6’-tetrachloro-1,1’3,3′-tetraethylbenzimid azolocarbocyanine iodide (JC-1), Hoechst33342, MitoTracker Red, Fluo-3 acetoxymethyl ester (Fluo-3/AM) and Rhod-2 acetoxymethyl ester (Rhod-2/AM; the dyes) were obtained from Molecular Probes (Carlsbad, CA). Polyclonal rabbit anti-human myocilin (N-15) antibody and polyclonal goat anti-human COX IV antibody were purchased from Santa Cruz Biotechnology (Santa Cruz, CA).

### Plasmid construction

The full-length myocilin cDNA clone has been described in our previous article and in another study [44,45]. Briefly, EcoRI and BamHI restriction enzyme cleavage sites were added to the 5′ and 3′ end of the myocilin cDNA by polymerase chain reaction (PCR). The obtained fragment was cloned into mammalian expression plasmid (pIRES and pIRES-EGFP; Clontech, Palo Alto, CA). We generated Pro370Leu mutations with the QuikChange site-directed mutagenesis kit (Stratagene, La Jolla, CA) according to the manufacturer's instructions. The specific PCR primers for Pro370Leu mutagenesis were as follows: 5′-CTA CCA CGG ACA GTT CCT GTA TTC TTG GGG TGG CTA-3′ and 5′-TAG CCA CCC CAA GAA TAC AGG AAC TGT CCG TGG TAG-3′. The sequence of the Pro370Leu myocilin mutant plasmid was verified using DNA sequencer to confirm correct insertion and the absence of undesirable mutations.

### Tissue procurement and cell culture

Procurement of the human tissue was performed according to the tenets of the Declaration of Helsinki. Normal human eyes were obtained from the Zhongshan Ophthalmic Center Eye Bank in Guangzhou, China as we mentioned in our previous studies [18,46]. The procurement of tissue was approved by the IRB Committee at the Sun Yat-sen University at Guangzhou. Normal TM cells were derived from 5 human donor eyes with no known ocular diseases. These eyes were obtained within 24 h after death and were dedicated for corneal transplantation. The ages of the donors ranged from 20 to 40 years.

The TM cultures were prepared as described before [18,46-49]. Briefly, human TM was carefully dissected from the anterior segments and the whole corneal layer of the donor eyes. The explants were placed in 24 well culture plates (Corning Costar, Cambridge, MA) containing Dulbecco’s modified Eagle’s medium (DMEM), which was supplemented with 15% fetal bovine serum, 2 mM L-glutamine, penicillin (100 U/ml), and streptomycin (100 μg/ml). Cells gradually migrated from the TM explants and formed a confluent monolayer in approximately 10–14 days. Fourth- or fifth-passage cells were used for all the studies described here. The TM cells were seeded at a density of 1×10^5^ cells per well using six-well tissue culture plates, and micrographs of the cultures were taken three days post seeding at approximately 80% confluency.

The identity of the TM cells has been characterized in our previous publications using immunocytochemical staining of fibronectin, laminin, and neuron-specific enolase [18,46], and by the increased myocilin expression after exposure to dexamethasone (10^–7^ M) for three days [50,51].

### Gene transfection

Fourth- or fifth-passage TM cells grown to about 80%–85% confluence were incubated for 6 h with 1 μg of pIRES-EGFP-WT, pIRES-WT, pIRES-EGFP-Pro370Leu, or pIRES-Pro370Leu myocilin expression plasmids in the presence of Lipofectamine (0.3% v/v; Invitrogen). The cells were then washed and cultured for another 24 h or 48 h. The efficiency of transfection was estimated by transiently transfecting the cells with pIRES-EGFP vector, and the percentage of EGFP-positive cells were analyzed using flow cytometry. The transfection efficiency was about 38%–46%. Also, TM cells transfected with the pIRES (Mock) plasmid was used as a negative control.

### Myocilin mRNA measurement

Myocilin mRNA expression was analyzed by reverse transcriptase-polymerase chain reaction (RT–PCR). Total mRNA from TM samples with or without transfection were isolated using RNeasy reagents (Qiagen, Valencia, CA) according to the manufacturer’s protocol. First-strand cDNA was synthesized using iScript cDNA synthesis kit (Bio-Rad, Hercules, CA). RT–PCR was performed using iTaq polymerase (Bio-Rad) at an annealing temperature of 59 °C for 30 cycles for both *myocilin* and Glyceraldehyde-3-phosphate dehydrogenase (*GAPDH*) primers. The primer sequences used in PCR reactions for the *myocilin* gene are (sense) 5′-CTG GAA ACC CAA ACC AGA GA-3′ and (antisense) 5′-AAA TTC GGG AAG CAG GAA CT-3′. *GAPDH* was used as the internal RNA loading control, and the samples where no reverse transcriptase (NRTs) was added to the PCR experiments were used as negative controls to confirm that the amplification was RNA dependent. PCR products were resolved by 1% agarose gel electrophoresis.

### Mitochondrial isolation and total cell lysate preparation

For mitochondrial protein extraction, mitochondria from TM cultures were isolated using a mitochondria isolation kit for cultured cells (Pierce Chemical Co., Rockford, IL). Briefly, after being transfected with pIRES-EGFP plasmid (Mock), pIRES-EGFP-WT myocilin, and pIRES-EGFP-Pro370Leu for 48 h, three 75 cm^2^ culture flasks of 85% confluent cells were homogenized in ice-cold buffer (225 mM mannitol, 75 mM sucrose, 1 mM EGTA, 5 mM HEPES, 2 mg/ml fat-free bovine serum albumin [BSA], and protease inhibitor cocktail [Roche Diagnostics, Branchburg, NJ]) with 30 up-and-down strokes using a motor-driven Teflon pestle that is tight-fitted to a Dounce homogenizer (Kontes Co., Vineland, NJ). The homogenate was centrifuged at 1,000x g for 10 min at 4 °C, and the resulting supernatant was layered onto 5 ml of 7.5% Ficoll medium (Sigma, St. Louis, MO) after being layered onto 5 ml of 10% Ficoll medium (0.3 M sucrose, 50 μM EGTA, and 10 mM HEPES) and centrifuged at 79,000x g for 30 min. Mitochondrial pellets were re-suspended in 500 μl of ice-cold mitochondrial buffer in a pre-weighed 0.5 ml tube and centrifuged. The supernatant was then discarded and the pellet weight estimated. The mitochondrial pellets were then lysed in 0.5 ml Cytobuster lysis buffer (Novagen, Madison, WI) and centrifuged, and protein concentrations were estimated using Dc Protein Assay Kit (Bio-Rad).

For total cellular protein preparation, TM cells transfected with pIRES plasmid (Mock), pIRES-WT myocilin, or pIRES-Pro370Leu mutant myocilin for 48 h were lysed in 0.5 ml Cytobuster lysis buffer and centrifuged, and protein concentrations were estimated using DC Protein Assay Kit.

### Western blot analysis

Forty micrograms of mitochondrial protein or total cellular protein was separated by SDS–PAGE and transferred onto nitrocellulose membranes (Bio-Rad). After blocking with 5% (w/v) dried milk, membranes were incubated with polyclonal rabbit anti-human myocilin (N-15) antibody (1:200) and polyclonal goat anti-human COX IV antibody (1:200; Santa Cruz Biotechnologies) at 4 °C overnight followed by washing and incubation with Horsheradish peroxidase (HRP)-conjugated goat anti-rabbit IgG (1:200; Bio-Rad) for 1 h at room temperature. Bound antibody was determined using the Bio-Rad ECL detection system.

### Morphological analysis of trabecular meshwork cultures after transfection

TM cells seeded at a density of 1×10^5^ cells per well in six-well plates were transfected for 24 h with pIRES-EGFP plasmid (Mock), pIRES-EGFP-WT myocilin, or pIRES-EGFP-Pro370Leu mutant myocilin. Phase contrast micrographs were taken to examine morphological changes after transfection.

Some of the cells were seeded in a 35 mm^2^ Petri dish and transfected with pIRES-EGFP plasmid (Mock), pIRES-EGFP-WT myocilin, or pIRES-EGFP-Pro370Leu mutant myocilin for 8 h. Cells were either stained with 1 μg/ml Hoechst33342 to illustrate the nuclei or labeled with 50 nM of MitoTracker Red to show the mitochondria. The staining pattern was visualized by Zeiss 100M confocal microscope (Carl Zeiss Jena GmbH, Jena, Germany).

### Measurement of reactive oxygen species

Cellular oxidative stress was determined by cytoplasmic ROS levels [52,53]. Briefly, cells transfected with pIRES plasmid (Mock), pIRES-WT myocilin, or pIRES-Pro370Leu mutant myocilin and non-transfected cells were trypsinized and re-suspended in freshly prepared H_2_-DCF-DA (0.4 μM) at a density of 2×10^6^ cells/ml at 37 °C in the dark for 30 min. H_2_-DCF-DA penetrates into cells and emits green fluorescence upon oxidation through a reaction with H_2_O_2_ and to a certain extent with NO. H_2_-DCF-DA-loaded cells were rinsed twice with PBS and analyzed immediately by flow cytometry at 488 nm excitation and 530 nm emission. Ten thousand cells were routinely collected, and data were expressed as the median fluorescence intensity in arbitrary units from the average of at least three repeated experiments.

### Measurement of calcium levels in the cytoplasm ([Ca^2+^]_c_) and mitochondria ([Ca^2+^]_m_) of trabecular meshwork cells

Changes in [Ca^2+^]_c_ and [Ca^2+^]_m_ were measured with the fluorescent probe fluo-3/AM (dissociation constant [K_d_] ~400 nM) and Rhod-2/AM (K_d_ ~570 nM), respectively, as described previously [46,54-56]. Fluorescence intensity of labeled cells was measured using flow cytometry.

The cells were cultured in six-well plates at a density of 1×10^5^ cells per well. After being transfected with pIRES plasmid (Mock), pIRES-WT myocilin, and pIRES-Pro370Leu mutant myocilin for 24 h, TM cells were loaded with either 1 μM fluo-3/AM for 30 min or 1 μM rhod-2/AM for 1 h. The cells were then trypsinized, washed twice with cold PBS, re-suspended in 200 μl PBS, and then immediately analyzed by flow cytometry at an excitation wavelength of 488 nm and an emission wavelength of 525 nm for fluo-3/AM and an excitation wavelength of 549 nm and emission wavelength of 581 nm for Rhod-2/AM. The fluorescent intensity of 10,000 labeled cells was routinely collected for each analysis and the data expressed as the median fluorescence intensity in arbitrary units from the average of at least three repeated experiments.

### Measurement of cellular ATP

ATP levels were determined using a luciferin/luciferase-based ATP assay. Briefly, cells grown in 24-well plates were transfected with pIRES plasmid (Mock), pIRES-WT myocilin, and pIRES-Pro370Leu mutant myocilin for 24 h. After removal of the treatments, the cell membrane was permeabilized by 100 μl of somatic cell ATP-releasing reagent (FL-SAR; Sigma-Aldrich Co., St Louis, MO) and was allowed to react with 100 μl of ATP Assay Mix Reagent (FLAA; Sigma-Aldrich Co.) containing luciferin and luciferase. After the 10 min incubation at room temperature, luminescence was measured with a 0.5 s integration time using a luminometer (Orion II Luminometer; Berthold Detection Systems, Oak Ridge, TN). The cellular ATP level was expressed as the percentage of luminescence intensity of normal human TM cells.

### Measurement of mitochondrial membrane potential

JC-1 was used to demonstrate the changes in the mitochondrial membrane potential (ΔΨm) in TM cells. JC-1 is a lipophilic and cationic dye, which permeates plasma and mitochondrial membranes. The dye fluoresces red when aggregates in healthy mitochondria with high membrane potential, whereas it appears in monomeric form and fluoresces green in mitochondria with diminished membrane potential. JC-1 was freshly diluted in serum-free DMEM to a final concentration of 1 μg/ml and was added to suspending transfected (pIRES, pIRES-WT myocilin, and pIRES-Pro370Leu mutant myocilin for 24 h) and non-transfected cells at a density of 2×10^6^ cells/ml. After incubation for 20 min at 37 °C in the dark, all samples were rinsed twice in PBS and analyzed immediately by flow cytometry at 488 nm excitation. Data were collected at 530 nm emission for green fluorescence of the JC-1 monomer on the filter 1 (FL1 detector) and at 590 nm for red fluorescence of JC-1 aggregates on the filter 2 (FL2 detector). Results were presented in relative aggregate to monomer (red:green) fluorescence intensity ratio. Cell numbers in quadrant 4 (Q4; green fluorescent only) were also recorded.

### Statistical analysis

For quantitative analyses, we conducted triplicate experiments on all five TM samples, and data from 15 trials were averaged. For qualitative analysis, all images were generated from the TM cell of one donor to represent similar changes observed in four other donors’ TM cells. Numerical results were analyzed using one-way ANOVA and expressed in mean±standard error (SE). Differences were considered statistically significant at p<0.05.

## Results

### Morphological changes of trabecular meshwork cells overexpressing Pro370Leu mutant myocilin

**Figure 1:**
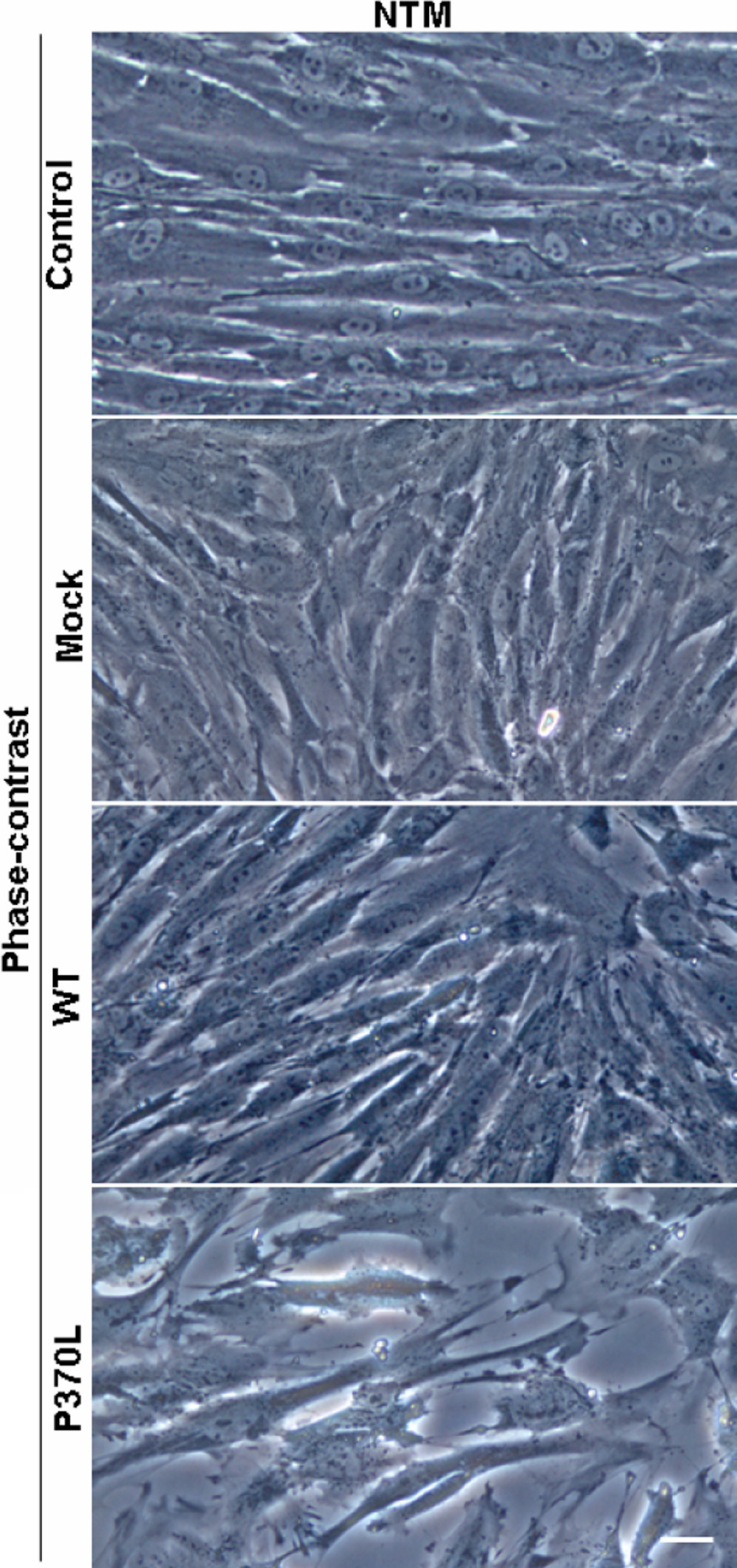
Morphology change of TM cells after Pro370Leu mutant myocilin transfection. Phase-contrast light micrographs show healthy looking TM cells in non-transfected (Control), pIRES transfected (Mock), or pIRES-WT myocilin transfected (WT) cultures. However, TM cells demonstrate an unhealthy, degenerative appearance after transfection with pIRES-Pro370Leu mutant myocilin (P370L) for 24 h. Fewer cells are seen in these cultures in part due to cell death and cell detachment. Scale bar=30 μm.

In phase-contrast micrographs, TM cells that were transfected with Pro370Leu mutant myocilin for 24 h looked unhealthy and exhibited cell shrinkage and shedding (Figure 1). Fewer cells were seen in these cultures in part due to cell death and cell detachment. TM cells with WT myocilin transfection maintained a relatively healthy appearance.

### Increased expression of myocilin after transfection

**Figure 2:**
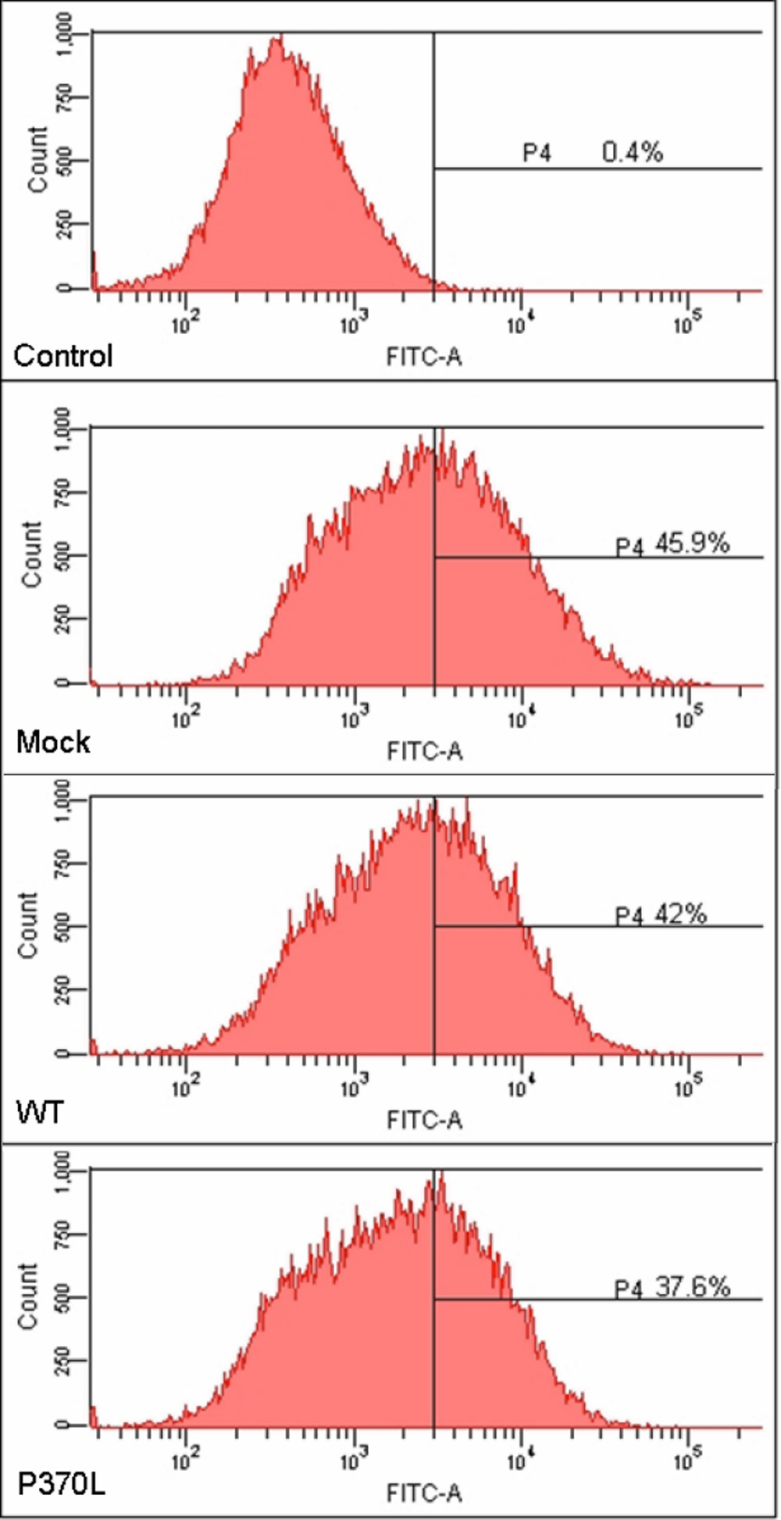
The efficiency of myocilin transfection. The efficiency of transfection was estimated in TM cells transfected with pIRES-EGFP (Mock), pIRES-WT myocilin (WT), or pIRES-Pro370Leu mutant myocilin (P370L) by counting the percentage of EGFP-positive cells using flow cytometry analysis. The non-transfected TM cells are used as the control. The transfection efficiency was about 38%–46%.

To determine the transfection efficiency, we transfected the TM cells with pIRES-EGFP plasmid and EGFP-positive cells were quantified using flow cytometry. The TM cells from all five donors showed similar responses to P370L myocilin transfection that EGFP-positive cells comprised 38%–46% of total cells in the culture (Figure 2).

**Figure 3:**
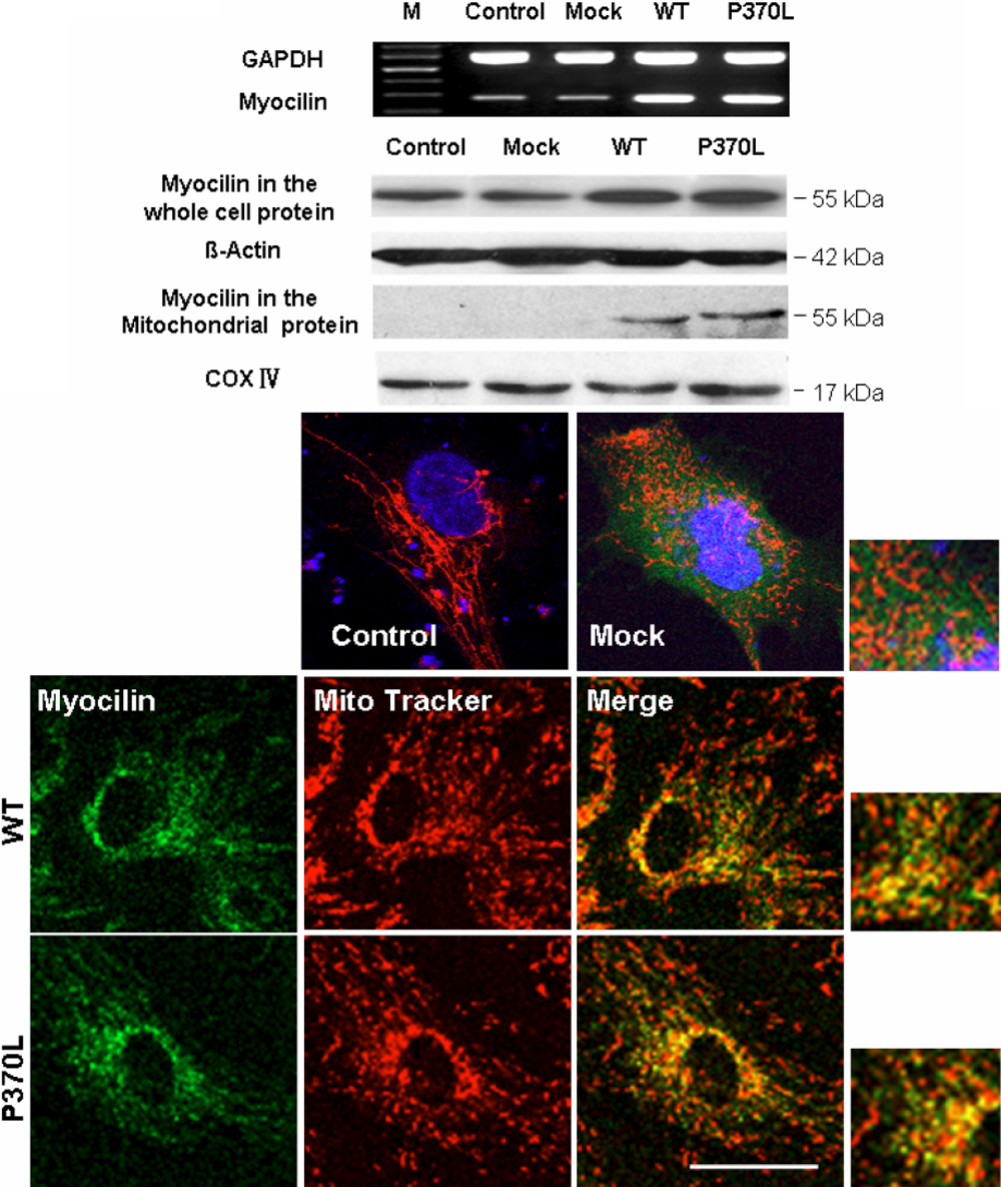
Increased expression of myocilin after transfection. RT- PCR and western blot show that transfection of WT or Pro370Leu (P370L) mutant myocilin increase both myocilin mRNA and protein levels in TM cells after 48 h. *GAPDH* was used as the internal loading control in RT–PCR while β-actin and cytochrome C oxidase (COX IV) were used as internal loading controls in western blot analysis for total cell lysate and mitochondrial lysate, respectively. Confocal images show myocilin co-localized with mitochondria. After transfection for 8 h, non-fixed TM cells were immunolabeled with MitoTracker Red, a mitochondria specific dye. Confocal microscopic analyses show that both pIRES-EGFP-WT myocilin (WT) and pIRES-EGFP-Pro370Leu mutant myocilin (P370L) transfected cells have overlapping EGFP staining (green fluorescence) and mitochondria staining (red fluorescence). However, the green fluorescence from pIRES-EGFP (Mock) is diffusive within the cells with no overlapping with mitochondrial staining. Our result indicates co-localization of myocilin and mitochondria in TM cells. Scale bar=30 μm.

We then transfected the TM cells with vector carrying WT or Pro370Leu mutant myocilin. After transfection for 48 h, the expression of WT or Pro370Leu was increased as determined by RT–PCR and western blot analysis (Figure 3).

### Myocilin co-localizes with mitochondria

The confocal images in Figure 3 show the mitochondrial staining with MitoTracker Red, a mitochondria specific dye, after transfection with plasmids for 48 h. From the confocal micrographs, both EGFP-WT and EGFP-Pro370Leu mutant myocilin transfected cells had demonstrated overlapping EGFP signals (green fluorescence) and mitochondrial staining (red fluorescence). In cells transfected with pIRES-EGFP (Mock), however, EGFP was expressed everywhere inside the cells, and no overlay with mitochondria was observed. This data indicated that myocilin co-localizatized with mitochondria in TM cells.

### Pro370Leu mutant myocilin induces reactive oxygen species generation, increases calcium levels in the cytoplasm ([Ca^2+^]_c_) and mitochondria ([Ca^2+^]_m_)

**Figure 4:**
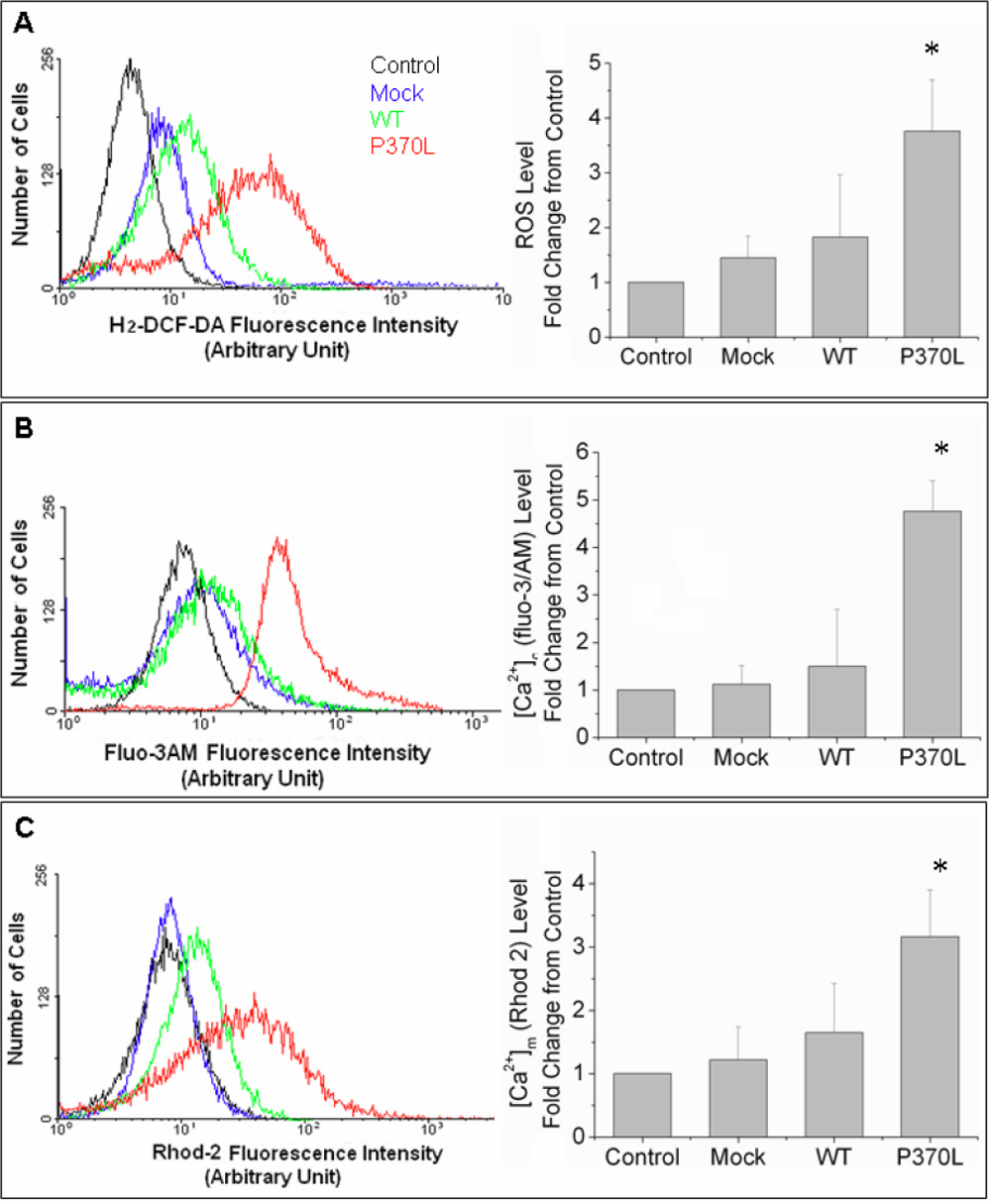
Pro370Leu mutant myocilin induces ROS generation and increases calcium levels in the cytoplasm and mitochondria in TM cells as estimated using flow cytometry. **A**: The oxidation sensitive fluorescence dye, H_2_-DCF-DA, was used to measure the ROS levels in TM cells. TM cells transfected with Pro370Leu (P370L) mutant myocilin transfection show 2.05 fold (±0.94) stronger H_2_-DCF-DA fluorescent intensity than those transfected with WT myocilin (WT), indicating an increase in ROS generation after Pro370Leu mutant myocilin transfection compared to WT myocilin transfection. **B**,**C**: The [Ca^2+^]_c_ and [Ca^2+^]_m_ indicators, fluo-3/AM and Rhod-2, were used to illustrated the free Ca^2+^ levels in the cytoplasm and mitochondria, respectively. There was a 3.17 fold (±0.64) increase in [Ca^2+^]_c_ and 1.92 fold (±0.74) increase in [Ca^2+^]_m_ after Pro370Leu mutant myocilin (P370L) transfection compared to WT myocilin transfection (WT). Data are expressed as fold changes in fluorescent levels of transfected TM cells to non-transfected TM cells (Control). Results are expressed as the mean±SE of three repeated experiments done in triplicate. An asterisk indicates a significant difference from non-transfected cells at p<0.05.

We next examined whether myocilin affects the function of mitochondria by measuring ROS, [Ca^2+^]_c_, and [Ca^2+^]_m_ levels in TM cells overexpressing WT or Pro370Leu mutant myocilin. In Figure 4, we present evidence determined by flow cytometry that Pro370Leu mutant myocilin significantly increased ROS generation (2.05±0.94 fold) as well as [Ca^2+^]_c_ (3.17±0.64 fold) and [Ca^2+^]_m_ (1.92±0.74 fold) levels in TM cells compared to WT myocilin.

### Pro370Leu mutant myocilin causes greater endogenous ATP and mitochondrial membrane potential reduction compared to wild type myocilin

**Figure 5:**
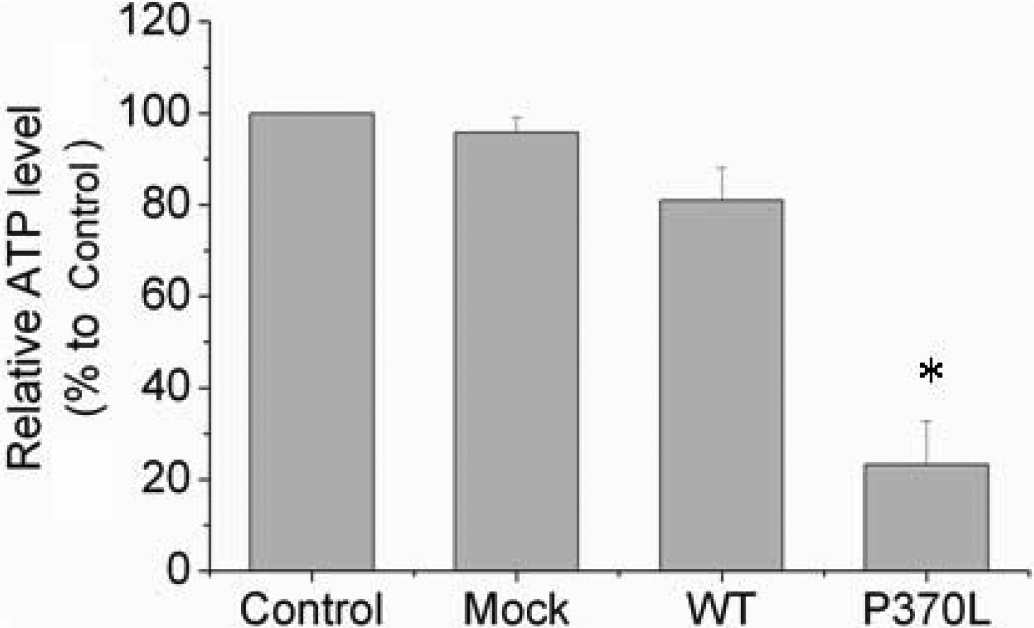
Pro370Leu mutant myocilin decreases ATP levels in TM cells. Luciferin/luciferase-based ATP assay indicates that the ATP level in TM with Pro370Leu mutant myocilin (P370L) transfection is 57.7% lower compared to WT myocilin transfection (WT). The ATP levels in non-transfected TM cells (Control) are arbitrarily defined as 100%. The results are expressed as a mean percentage of the ATP levels to the control, error bars represent SE of three repeated experiments done in triplicate. An asterisk indicates a significant difference from non-transfected cells at p<0.05.

**Figure 6:**
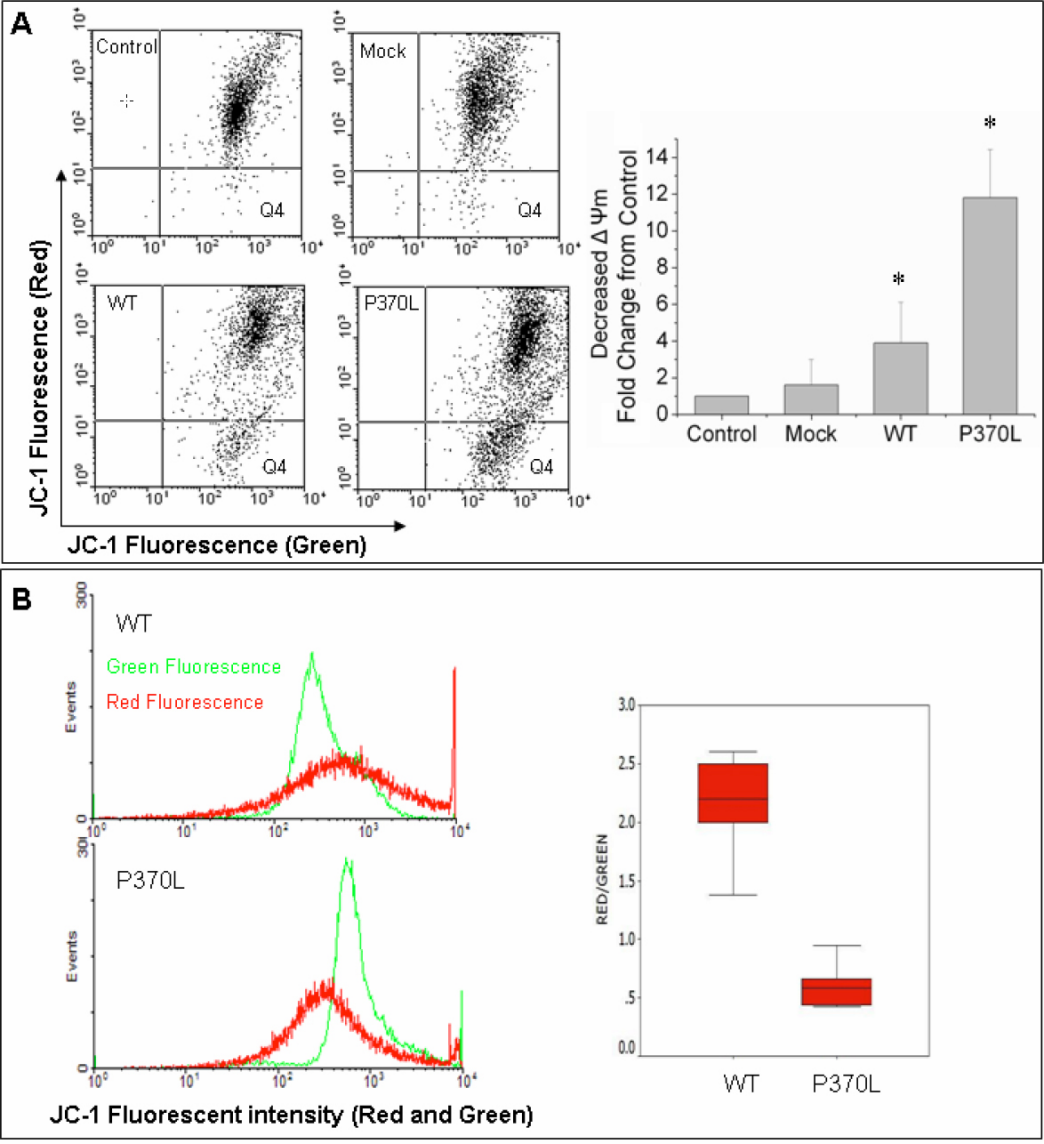
Pro370Leu mutant myocilin further decreases mitochondrial membrane potential in TM cells. ΔΨm was examined using flow cytometry with the fluorescence indicator, JC-1. The dye fluoresces red when aggregates in healthy mitochondria with high membrane potential, whereas it appears in monomeric form and fluoresces green in mitochondria with diminished membrane potential. The cells number in the Q4 area (green fluorescence) indicates the number of cells with low ΔΨm. The histogram shows the relative decrease of ΔΨm to the non-transfected cell (Control). **A**: The ΔΨm is 3.03 fold (±0.90) lower in TM cells transfected with Pro370Leu mutant myocilin (P370L) compared to TM cells transfected with WT myocilin (WT) while there is a 2.44 fold (±0.87) reduction in WT compared to Mock. **B**: Pro370Leu mutant myocilin transfected TM cells have a significantly lower red to green ratio, and more cells show green fluorescence (cells in Q4 area), indicating Pro370leu mutant myocilin causes a greater mitochondria membrane depolarization. Results are expressed as the mean fold decrease in relative aggregate, a monomer (red:green) ratio of transfected TM to non-transfected TM cells. The error bars represent SE of three repeated experiments done in triplicate. An asterisk indicates a significant difference from non-transfected cells at p<0.05.

ATP depletion and mitochondrial depolarization (decrease in ΔΨm) are the major events in mitochondrial dysfunction. Figure 5 shows that there was a decrease in endogenous ATP levels in TM cells after Pro370Leu mutant myocilin transfection compared to those with WT myocilin transfection (p<0.05). In addition, TM cells after Pro370Leu mutant myocilin transfection had a lower ΔΨm compared to those with WT myocilin transfection (p<0.05; Figure 6).

## Discussion

Recently, we reported a Pro370Leu mutation of the myocilin gene in a large Chinese autosomal dominant JOAG family (GZ.1) encompassing 56 family members with 19 of them exhibiting JOAG. All these JOAG-affected family members present similar clinical symptoms of high IOP, typical glaucomatous cupping of the optic disc, and a thinner nerve fiber layer and are unresponsive to standard pharmacological treatments [25]. From these patients, we were the first to report that Pro370Leu mutant myocilin enhances ER stress response in human TM cells [45], but how Pro370Leu mutant myocilin affects mitochondrial function is still unclear. Another study has indicated a mutation of this gene causes apoptosis in TM cells [24]. Here, we illustrate the interaction of Pro370Leu myocilin with mitochondria and proposed a possible mechanism that may lead to TM cell damage.

In this study, we provide evidence of an association between myocilin and mitochondria and found that TM cells expressing Pro370Leu mutant myocilin exhibit mitochondrial dysfunction that is not seen in cells expressing WT myocilin. Sakai et al. showed that the myocilin enters the mitochondria and interacts with the mitochondrial membranes [42]. His findings corroborates results from immunogold labeling, fluorescence labeling, and subcellular fractionation experiments that myocilin is associated with mitochondria in TM cells [39-42]. However, myocilin localization in the mitochondria is not observed in corneal fibroblasts or in the mouse liver. The regulatory mechanisms of such cell-specific localization of myocilin require further investigation, but this phenomenon makes TM cells more sensitive to any undesirable effects of myocilin mutations on the mitochondria.

The fact that both WT and Pro370Leu mutant myocilin sit on the mitochondria raises the question whether these two proteins have different effects to the function of mitochondria. Because growing evidence has shown the relationship between oxidative stresses and exacerbated glaucomatous conditions [17,18,57,58], we compared ROS levels in the normal human TM cells and those cells after WT myocilin or Pro370Leu mutant myocilin transfection. We found that the endogenous ROS level is higher in the TM cells following Pro370Leu mutant myocilin transfection compared to those with WT myocilin transfection, indicating that increased oxidative stress is associated with the pathogenesis of Pro370Leu mutant myocilin in POAG patients. There is a general consensus that cumulative oxidative damage is responsible for general aging [59-62]. Although many individuals with POAG were young adults in the GZ.1 family [25], the higher ROS levels in the TM cells with Pro370Leu mutant myocilin gene could possibly accelerate the aging process in this region, which may in part contribute to the progression of POAG.

Myocilin increases both [Ca^2+^]_c_ and [Ca^2+^]_m_, possibly through the disregulation of calcium channels. Calcium-dependent contractibility of TM cells control outflow of the aqueous humor, which maintains IOP. Failure in closing the L-type calcium channel leads to an excessive influx of Ca^2+^, causing membrane depolarization, TM contraction, reduced outflow, and IOP elevation. Though a direct interaction between Pro370Leu mutant myocilin and the L-type calcium channel is not conclusive from our data, it is a possible explanation to our observation.

On top of its ATP generation ability, the mitochondria also plays a part in modulating the amplitude and spatiotemporal organization of Ca^2+^ signals through rapidly accumulating and releasing Ca^2+^ [63,64]. However, excessive cytoplasmic Ca^2+^ leads to mitochondrial Ca^2+^ overload, which triggers ROS overproduction, mitochondrial membrane depolarization, and ATP production inhibition, all hallmark events of mitochondrial dysfunction and eventual apoptosis [65,66].

Studies showed that myocilin is imported into the mitochondria and may target the inner and outer membranes and inter-membrane space [42]. However, the structure of myocilin does not seem to have a typical NH_2_-terminal mitochondrial-targeting presequence. Myocilin is predicted to contain a potential mitochondrial transit peptide at its NH_2_-terminus (amino acid residues 1–47) using the PCGene program (IntelliGenetics Inc., Mountain View, CA) base on the charge transition and R(2) weak rule [67]. Also, both the NH_2_- and COOH-termini of myocilin contain a lysine- and arginine-rich mitochondrial tethering domain (amino acid residues 33–46 and 460–504) [68]. Like the predictions for other mitochondrial outer membrane proteins, some data showed the α-helical transmembrane segment of the NH_2_-terminal is the main contributor for myocilin to incorporate into mitochondrial membranes, while the beta-sheet segment at the COOH-terminus play a comparatively minor role [42,69-71]. However, the exact targeting sequence for mitochondrial translocation of myocilin is still unclear.

Myocilin appears to be imported into the mitochondria with the aid of a high molecular weight translocator complex in the outer and inner mitochondrial membranes [42], which has been demonstrated with other mitochondrial proteins [70,72-75]. Mutation in myocilin alters the protein structure and conformation [76-78]. Since Pro370Leu is located within the highly conserved Olfactomedin (OLF)-domain at the COOH-terminus of myocilin [79], it is possible that the mutation changes the protein structure and conformation, which negatively impacts the mitochondrial inner and outer membrane functions. The alteration in mitochondria may subsequently lead to differential effects on ROS and ATP generation, Ca^2+^ levels in cytoplasm and mitochondria, and ΔΨm in TM cells.

In conclusion, we present strong evidence of the association of myocilin with mitochondria. Increased generation of ROS, decreased ATP production, decreased ΔΨm, and increased Ca^2+^ levels in both the cytoplasm and mitochondria in TM cells with Pro370Leu mutant myocilin gene transfection indicate that Pro370Leu mutant myocilin can affect mitochondria functions. The mutation increases vulnerability in the mitochondria to various cellular injuries and disables normal human TM cells functions, which may eventually contribute to the failure of the TM to control IOP. New insights on the protective effects of antioxidants and mitochondria permeability transition inhibitors to the health of TM cells [18,46] suggest that there are POAG treatment possibilities that are worthy of further investigation.
